# Association between dietary B vitamins intake and age-specific blood pressure: A cross-sectional study in American adults

**DOI:** 10.1371/journal.pone.0335306

**Published:** 2025-10-22

**Authors:** Xianfeng Li, Zhiqiang Nie, Fumei Zheng, Yuandi Lei, Shuqi Chen, Shan Liu

**Affiliations:** 1 Longgang District Maternity & Child Healthcare Hospital of Shenzhen City (Affiliated Shenzhen Women and Children’s Hospital (Longgang) of Shantou University Medical College), Shenzhen, China; 2 Hypertension Research Laboratory, Global Health Research Center, Guangdong Provincial People’s Hospital (Guangdong Academy of Medical Sciences), Southern Medical University, Guangzhou, China; 3 Shenzhen Longgang District Sixth People’s Hospital, Yicheng Scenic Community Health Service Center, Shenzhen, China; 4 School of Public Health, Kunming Medical University, Kunming, China; Southern Illinois University Carbondale, UNITED STATES MINOR OUTLYING ISLANDS

## Abstract

**Objective:**

To explore the relationship between B vitamins and blood pressure across distinct age groups.

**Methods:**

We analyzed 3654 participants aged ≥20 years after excluding pregnant/lactating individuals and those with incomplete data. B-vitamin intake was calculated from two 24-hour dietary recalls and supplement surveys. Exposure variables included 7 kinds of B vitamins intake, while outcomes comprised hypertension prevalence, systolic and diastolic pressure. The covariates include basic demographics, lifestyle factors, chronic Comorbidities, and nutrient intake. Based on the cross-sectional study, the statistical analyses incorporated NHANES sampling weights. Weighted logistic regression evaluated hypertension association, while linear regression assessed systolic/diastolic pressure differences. RCS model explored nonlinear dose-response relationships.

**Results:**

Weighted logistic regression and RCS model revealed age-specific and nonlinear characteristics in the b vitamin–blood pressure relationship. Vitamin B1 was negatively correlated with hypertension in ≥60 years old (Q4: Q1 OR (95%CI) =0.27 (0.08, 0.91)). Vitamin B2 was negatively correlated with hypertension in total population at Q4 (OR (95%CI) =0.39 (0.15, 0.99)), and in aged 40–59 years, OR (95%CI) =0.52 (0.33, 0.83). Choline was positively correlated with hypertension in the total population (OR (95%CI) =1.30 (1.08, 1.58)) but negatively correlated with ≥60 years old (OR (95%CI = 0.73 (0.56, 0.95)). Vitamin B12 shown positive associations with hypertension in the total population, 20–39 years old and ≥60 years old OR (95%CI) =1.39 (1.13, 1.71), 1.82 (1.23, 2.69), and 1.63 (1.04, 2.54), respectively. For diastolic pressure, vitamin B1, B2, niacin, B6, and folate displayed significant negative associations in the total population and ≥60 years old. Vitamin B2 was also negatively associated in 40–59 years old. Vitamin B12 exhibited a negative association with diastolic pressure in ≥60 years old. Weight RCS analysis revealed the linear or nonlinear relationships between specific B vitamins and hypertension and systolic/diastolic pressure, with age stratification improving the discernment of these associations.

**Conclusions:**

Associations between B vitamins intake and hypertension/blood pressure exhibited age-dependent variations. Age-specific considerations are essential for optimizing B vitamins supplementation or restriction.

## 1. Introduction

Hypertension is defined as blood pressure (BP)≥140/90 mm Hg [1], and the 2017 ACC/AHA guidelines defined stage 1 hypertension in special populations as blood pressure ≥130/80 mm Hg [2], which is a major preventable risk factor for cardiovascular disease and all-cause death worldwide [3,4]. Data from 2019 shown that 626 million (584–668) women and 652 million (604–698) men aged 30–79 years had hypertension [5]. A large number of studies have shown that the occurrence of hypertension was related to unhealthy eating habits, such as excessive drinking, heavy salt diet and low potassium diet, which are all risk factors for the occurrence and development of hypertension [6–8]. At the same time, healthy dietary patterns such as Dietary Approaches to Stop Hypertension (DASH), Mediterranean Diet and Plant-Based Diet can reduce blood pressure [9–11]. Lowering blood pressure has been shown to reduce the incidence of stroke, heart attack and heart failure [12]. However, emerging evidence suggests that micronutrient deficiencies, particularly b-vitamin deficiencies, may play an under-recognized role in blood pressure regulation through mechanisms such as homocysteine metabolism [13], oxidative stress [14] and endothelial function [15].

B-vitamins are water-soluble vitamins that are indispensable for maintaining human function and metabolism, and the human cannot manufacture and synthesize by ourselves, and must be supplemented [16]. Studies in recent years have found B vitamins were uniquely positioned in blood pressure regulation due to their dual role in one-carbon metabolism and vascular homeostasis. But the association between B vitamins intake and blood pressure contraction and diastole are still controversial [17].

For example, recent study has found that vitamin B1 can reduce the risk of hypertension in people [18]. High intake of folate was found to be associated with decreased blood pressure levels in children [19], but positively correlated in the adolescent population [20]. However, a 20-year prospective cohort study demonstrated an inverse association between folate intake and the risk of hypertension among young American adults [21]. The antihypertensive effect of vitamin B12 was also controversial [22,23]. This might be due to the age-dependent changes in the use of folate and vitamin B12 to clear homocysteine (Hcy), and reflects the interaction of bioavailability thresholds or age-related methylation capabilities [24,25].

Furthermore, Study also has shown that dietary niacin can reduce the risk of hypertension in Chinese people, but there was a J-shaped association, and a positive correlation after the inflection point [26]. There was a linear association between niacin intake and hypertension in people >40 years old in the US population [27]. These inconsistencies likely stem from two methodological limitations: (1) in prior linear model failure to account for non-linear dose-response relationship using restricted cubic spline (RCS) analysis, and (2) inadequate stratification by age despite known declines in B-vitamins absorption.

The metabolism of B vitamins were closely related to a variety of lifestyle factors, among which smoking and excessive drinking can significantly reduce their bioavailability through different mechanisms. For example, smoking leads to a decrease in the circulating concentrations of vitamin B6 (pyridoxal −5’ -phosphate, PLP) [28] and folate by inducing oxidative stress and inhibiting intestinal absorption [29]. Excessive drinking can increase the risk of vitamin B12 deficiency by damaging the function of the gastric mucosa, inhibiting liver storage and accelerating the excretion of vitamin B12 [30,31]. Therefore, when conducting research related to dietary B vitamins intake, it is very necessary to incorporate lifestyle behavior patterns into the co-variables of the study in order to better expose the association between vitamin intake and outcomes.

Previous studies have typically regarded B vitamins as a homogeneous group or focused on isolated nutrients, neglecting their synergistic/antagonistic effects. Therefore, we aim to create a cross-sectional study on the association between dietary B vitamins and hypertension/blood pressure using data from the National Health and Nutrition Examination Survey (NHANES), through age-stratified design for the research subjects. To reveal the bidirectional effect between vitamins and blood pressure, understand the dose-effect relationship between B vitamins intake and blood pressure, thereby clarifying whether the obvious contradiction in previous literature is true biological heterogeneity or methodological human factors, providing the public with a more reasonable intake and maintaining people's health.

## 2. Method

### 2.1 Study population

**Fig 1:**
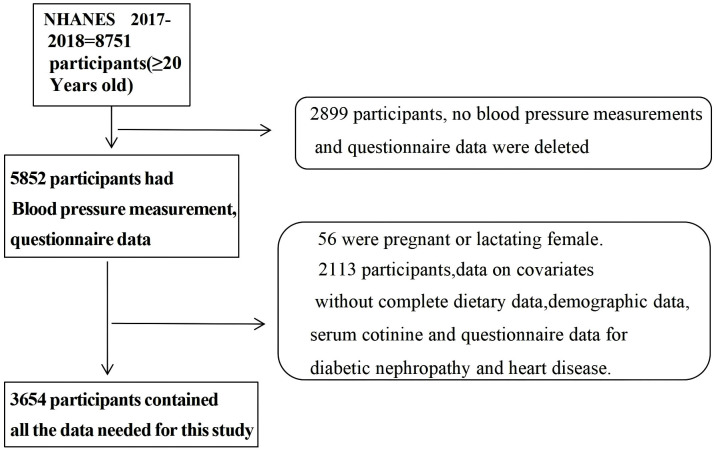
Participant flowchart, NHANES 2017–2018.

The NHANES is a cross-sectional study designed to evaluate the health and nutritional status of children and adults across the United States. Details of the study have been previously published in NHANES [32]. National Center for Health Statistics (NCHS) Ethics Review Board (ERB) ensures protections of all human participants in NCHS studies and surveys. The NHANES protocol has been reviewed and given ethical approval by the NCHS ERB. All participants provided written informed consent prior to their involvement (https://www.cdc.gov/nchs/nhanes/about/erb.html). In this study, due to the distinct vitamin intake patterns of pregnant and lactating women compared to the general population, these individuals were excluded. The complete data set of individuals with full records of three blood pressure measurements, hypertension questionnaire data, and dietary nutrition survey data was retained. After excluding individuals with missing data for all variables (including hypertension, systolic and diastolic blood pressure, age ≥ 20 years, dietary sodium intake, potassium intake, energy intake, carbohydrates intake, fat intake, protein intake, B vitamins intake, diabetes, cardiovascular disease (CVD), gender, kidney disease, body mass index (BMI), alcohol consumption, exercise, poverty to income ratio (PIR), education level, race, serum cotinine, marital status, use of hypertension medications and doctor told you that you had hypertension), the study included 3654 participants. The screening process for the study population is illustrated in Fig 1. In this study, all the research information can get from the following URL: https://wwwn.cdc.gov/nchs/nhanes/continuousnhanes/default.aspx?BeginYear=2017.

### 2.2 Measurement of blood pressure

After the participants had rested for 5 min and the participant's maximum inflation level (MIL) has been determined, and seated BP was measured by trained research staff. Three consecutive BP readings were obtained for each NHANES participant (https://wwwn.cdc.gov/nchs/nhanes/). If a BP measurement is interrupted or incomplete, a fourth attempt will be made. According to The American College of Cardiology/American Heart Association Guideline for the Prevention, Detection, Evaluation, and Management of High Blood Pressure in Adults (2017 Guideline) [2], the blood pressure of the population was defined as normal: the average systolic pressure <140 mm Hg and diastolic pressure <90 mm Hg of the three measurements, and “Ever told you had high blood pressure” answered “No” in the questionnaire data. Systolic pressure ≥140 mm Hg or diastolic pressure ≥90 mm Hg of the three measurements, or “Ever told you had high blood pressure” to answer “Yes” were hypertension.

### 2.3 Dietary nutrients intakes

Dietary intake data were collected by trained interviewers and used to estimate the types and amounts of foods consumed by participants in the 24 hours prior to the interview, and to estimate energy, nutrients, and other food components consumed from these foods and beverages. Dietary nutrient and energy intakes were estimated using the USDA Dietary Research Food and Nutrition Database (FNDDS). In the NHANES, the first meal recall interview was collected in person at a mobile screening center (MEC) and the second interview was collected over the phone 3 to 10 days later. In this study, we took a “Total Nutrient Intakes, First Day”, “Total Nutrient Intakes, Second Day”, “Total Dietary Supplements, First Day” and “Total Dietary Supplements, Second Day” were obtained. We first select the population with the “Total Nutrient Intakes, First Day” data, and based on that data, if the participant's “Total Nutrient Intakes, Second Day” data, we calculated total dietary nutrient intakes as (Day 1 intakes + Day 2 intakes)/2, and retained day 1 dietary data if day 2 dietary data was missing. Dietary supplement intake was calculated by comparing data from “Total Dietary Supplements, First Day” and “Total Dietary Supplements, Second Day”, using either results if only one survey was available, or calculating the mean if results were available from both surveys. Finally, the sum of total dietary nutrient intake and total dietary nutrient supplement intake was used as the dietary intake of B vitamins in this study. More detailed information can be found in the NHANES website: https://wwwn.cdc.gov/nchs/nhanes/search/datapage.aspx?Component=Dietary&CycleBeginYear=2017.

### 2.4 Assessment of covariates

Covariance was mainly for the demographic data, questionnaire data, laboratory data, and dietary survey data. It included gender, age group (20–39, 40–59, ≥  60), education level (less than 9th grade, 9–12th grade/High school graduate/GER or equivalent, college or AA degree/College graduate or above), race (Mexican American, other Hispanic, non-Hispanic White, non-Hispanic Black, other Race – Including Multi-Racial), PIR (<1, 1–2.5, ≥ 2.5), sedentary time(≤4, 4–8, ≥ 8 hours/day), alcohol consumption as “yes” or “no” based on the answer” yes” or “no” to “Ever had a drink of any kind of alcohol”, BMI (normal or low(14.8–24.9), overweight(25.0–29.9), obesity(≥30.0)), marital status (unmarried, married) and serum cotinine. According to the questionnaire data “Ever told you have diabetes/pre-diabetes”, diabetes was divided into normal, pre-diabetes, and diabetes. Participants’ CVD was determined based on their responses to “Ever told you have congestive heart failure/coronary heart disease/angina/heart attack/stroke?” (yes, no). According to “Ever told you had weak/failing kidneys?”, kidney disease was defined into yes or no. Energy intake, protein intake, carbohydrates intake, sodium intake, potassium intake, and fat intake were calculated in the same way as the above B vitamins.” Taking prescription for hypertension” was used to define the variable “Taking anti-hypertensive drugs”, this covariate was only included in the analysis of B vitamins and systolic and diastolic pressure. The PIR was calculated as the ratio of family income to the federal poverty level, adjusted for family size, which means a higher ratio indicates a higher income level relative to the poverty line [33].

### 2.5 Statistical analysis

According to the NHANES Analysis Guidelines, the analysis included sample weighting to account for complex survey design. To ensure nationally representative estimates, we applied the 2-year MEC exam weight (WTMEC2YR). The continuous variables in the baseline table are expressed by the median (P25, P75), and the categorical variables are expressed by the number of use cases n and percentage. For categorical variables, Chi-square tests were used to determine whether there were differences between age groups. Normality tests are performed on the continuous data. If the data conform to normal distribution and homogeneity of variance, ANOVA was used to test whether there were differences between different age groups. If the data distribution was not normal, the Spearman rank correlation coefficient was used to analyze the difference between the age groups.

We used weighted logistics and linear regression analyses to assess the association between dietary B-vitamins intake and hypertension prevalence or systolic and diastolic pressure. Given that previous studies have shown that dietary vitamin intake may not necessarily have a linear relationship with systolic and diastolic pressure. Following the common practice of epidemiology and similar study using NHANES data, we prioritized weighted restricted cubic spline (RCS) to avoid over-fitting while retaining sufficient flexibility to capture potential nonlinear relationships [34–36]. The use of sections 3–5 is widely recommended and considered sufficient for most applications in biomedical research. We compared the models in sections 3, 4, and 5 using the Akaike Information Criterion (AIC) and found that for most combinations of B vitamins and hypertension, systolic and diastolic blood pressure, a model with 4 sections achieved a good balance between goodness of fit and model complexity. We used 4-node analysis for all weighted RCS to explore the no-linear relationship between systolic and diastolic pressure and dietary B-vitamins intake in adults.

The distribution of intake of B vitamins and other nutrients were usually slanted, and they were converted logarithmically so that they were transformed into normally distributed data and treated as continuous variables. All analyses were adjusted for age group, alcohol consumption, BMI, education level, race, marital status, serum cotinine, PIR, gender, exercise, diabetes status, CVD, kidney disease, taking anti-hypertensive drugs, energy intake, fat intake, protein intake, sodium intake, potassium intake, and carbohydrate intake. We mainly analyzed Stata 15 and R 4.0.1 software. Zstats 1.0 platform (www.zstats.net) was using for generating statistical tables. A two-sided P < 0.05 considered statistically significant.

### 2.6 Addressing potential sources of bias

The complex sampling design of NHANES ensures nationwide representatives, but non-response situations may introduce biases. We adopted survey weights and included the population with complete datasets in the study to mitigate the impact of selection bias. The dietary recall data have memory biases. Therefore, we used the average of two recall data to improve accuracy and adjusted the total energy intake to reduce measurement errors. By selecting the average of three blood pressure measurements as the participant's blood pressure value, we tried to minimize the differences. Before the study began, relevant variables were selected based on the identified variables that might affect blood pressure (such as age, BMI, sodium and potassium intake). After adjusting for these factors, multiple regression analysis and the RCS model were employed for the study to reduce confounding bias.

## 3. Result

### 3.1 Demography

A total of 3654 people over the age of 20 were included in the study, including 1811 men and 1714 with hypertension were included. As Table 1 shown, in addition to diastolic pressure, niacin, choline and alcohol consumption, there were statistical differences among different age groups, *P-value* < 0.05 (Table 1).

**Table 1:** Demographic data of the NHANES population over 20 years old, 2017-2018.

	Total population	20-39 years old	40-59 years old	≥60 years old	*P-value*
**Number, N (%)**	3654(100)	1070(29.28)	1184(32.40)	1400(38.31)	
**Hypertension, N (%)**					
No	1940(53.09)	895(46.13)	656(33.81)	389(20.05)	<0.001*
Yes	1714(46.91)	175(10.21)	528(30.81)	1011(58.98)	
**Diastolic pressure (mm Hg), M (Q₁, Q₃)**	73.33 (65.33,82.67)	70.67(64.00,78.00)	76.67 (70.67,84.67)	70.67 (63.33,78.67)	0.089
**Systolic pressure (mm Hg), M (Q₁, Q₃)**	124.00 (113.33,137.33)	114.67 (106.67,123.33)	124.00 (114.00,135.33)	133.33 (121.33,148.00)	<0.001*
**Sodium intake (mg/day), M (Q₁, Q₃)**	3085.00 (2244.50,4119.00)	3410.75 (2454.00,4459.00)	3119.25 (2272.25,4231.75)	2860.75 (2098.50,3708.75)	<0.001*
**Potassium intake (mg/day), M (Q₁, Q₃)**	2390.00 (1768.62, 3133.75)	2312.25 (1708.88,3094.00)	2435.75 (1813.38,3198.62)	2409.50 (1785.75,3116.50)	0.025*
**Energy intake(kcal/day), M (Q₁, Q₃)**	1912.25 (1446.00,2510.50)	2044.00 (1545.50,2666.50)	1970.00 (1468.25,2597.75)	1792.25 (1374.50,2310.75)	<0.001*
**Protein intake(gm/day), M (Q₁, Q₃)**	72.98 (54.23,96.04)	77.52 (56.64,101.89)	74.88 (54.46,98.78)	69.14(52.23,89.75)	<0.001*
**Fat intake (gm/day), M (Q₁, Q₃)**	76.08(54.42,103.10)	81.24 (57.82,109.06)	76.61(54.13,104.89)	72.84(52.12,97.82)	<0.001*
**Carbohydrates intake (gm/day), M (Q₁, Q₃)**	224.02 (166.93,295.76)	236.48 (173.46,315.05)	233.58 (170.70,309.70)	210.26(158.64,271.25)	<0.001*
**Vitamin B1(mg/day), M (Q₁, Q₃)**	1.68(1.13,2.60)	1.57(1.11,2.38)	1.68(1.12,2.58)	1.77(1.16,2.82)	<0.001*
**Vitamin B2(mg/day), M (Q₁, Q₃)**	2.05(1.39,3.19)	1.89(1.35,2.85)	2.05(1.37,3.20)	2.17(1.42,3.35)	<0.001*
**Niacin(mg/day), M (Q₁, Q₃)**	26.58(18.35,39.24)	26.47(18.58,38.84)	26.72(18.58,38.95)	26.54(17.95,39.77)	0.578
**Vitamin B6(mg/day), M (Q₁, Q₃)**	2.21(1.40,3.93)	2.12(1.39,3.50)	2.22(1.39,3.89)	2.25(1.42,4.39)	0.003*
**Folate (mcg/day), M (Q₁, Q₃)**	538.50 (312.00,979.00)	494.00 (300.00,831.00)	536.50 (326.00,971.50)	581.50 (311.00,1037.00)	<0.001*
**Choline (mg/day), M (Q₁, Q₃)**	296.58 (208.90,412.25)	297.35 (206.60,419.25)	291.93 (205.55,421.40)	299.68(212.08,404.00)	0.859
**Vitamin B12 (mcg/day), M (Q₁, Q₃)**	5.29(2.72,13.32)	4.67(2.59,9.21)	5.03(2.65,12.38)	6.38(2.87,27.61)	<0.001*
**Serum cotinine (ng/mL), M (Q₁, Q₃)**	0.032(0.011,5.78)	0.079(0.016,53.70)	0.034(0.011,25.00)	0.020(0.011,0.197)	<0.001*
**BMI, N (%)**					
Normal or low	925(25.31)	359(38.81)	258(27.89)	308(33.30)	<0.001*
Overweight	1147(31.39)	254(22.14)	388(33.83)	505(44.03)	
Obese	1582(43.30)	457(28.89)	538(34.01)	587(37.10)	
**Gender, N (%)**					
Male	1811(49.56)	521(28.77)	561(30.98)	729(40.25)	0.047*
Female	1843(50.44)	549(29.73)	623(33.80)	671(36.41)	
**Race, N (%)**					
Mexican American	458(12.53)	153(33.41)	169(36.90)	136(29.69)	<0.001*
Other Hispanic	320(8.76)	84(26.25)	102(31.87)	134(41.88)	
Non-Hispanic White	1417(38.78)	385(27.17)	395(27.88)	637(44.95)	
Non-Hispanic Black	805(22.03)	238(29.57)	251(31.18)	316(39.25)	
Other Race – Including Multi-Racial	654(17.90)	210(32.11)	267(40.83)	177(27.06)	
**PIR, N (%)**					
< 1	637(17.43)	216(33.91)	220(34.54)	201(31.55)	<0.001*
1-2.5	1382(37.82)	411(29.74)	381(27.57)	590(42.69)	
≥ 2.5	1635(44.75)	443(27.09)	583(35.66)	609(37.25)	
**Educational level, N (%)**					
Less than 9th grade	241(6.60)	35(14.52)	75(31.12)	131(554.36)	<0.001*
9–12th grade/High school graduate/GER or equivalent	1284(35.14)	372(28.97)	387(30.14)	525(10.89)	
College or AA degree/College graduate or above	2129(58.26)	663(31.14)	722(33.91)	744(34.95)	
**Sedentary time (hours/day), N (%)**					
≤ 4	1622 (44.39)	484 (45.23)	532 (44.93)	606 (43.29)	0.059
4-8	993 (27.18)	278 (25.98)	297 (25.08)	418 (29.86)	
≥ 8	1039 (28.43)	308 (28.79)	355 (29.98)	376 (26.86)	
**Alcohol consumption, N (%)**					
Yes	3329(91.11)	986(29.62)	1077(32.35)	1266(38.03)	0.323
No	325(8.89)	84(25.85)	107(32.92)	134(41.23)	
**Marital status, N (%)**					
Unmarried	1799(49.23)	678(37.69)	504(28.02)	617(34.30)	<0.001*
Married	1855(50.77)	392(21.13)	680(36.66)	783(42.21)	
**Diabetes status, N (%)**					
Normal	2682(73.40)	976(36.39)	881(32.89)	824(30.72)	<0.001*
Pre-diabetes	395(10.81)	72(18.23)	144(36.46)	179(45.32)	
Diabetes	577(15.79)	22(3.81)	158(27.38)	397(68.80)	
**CVD, N (%)**					
No	3202(81.63)	1054(32.92)	1092(34.10)	1056(32.98)	<0.001*
Yes	452(12.37)	16(3.54)	92(20.35)	344(76.11)	
**Kidney disease, N (%)**					
No	3506(95.95)	1055(30.09)	1154(32.92)	1297(36.99)	<0.001*
Yes	148(4.05)	15(10.14)	30(20.27)	103(69.59)	
**Taking anti-hypertensive drugs, N (%)**					
No	2378(65.08)	989(41.59)	796(33.47)	593(24.94)	<0.001*
Yes	1276(34.92)	81(6.35)	388(30.41)	807(63.24)	

Note:

1.* It means that there is a significant difference in the *P value.*

2.CVD: Cardiovascular disease, BMI: body mass index, PIR: poverty to income ratio.

3.The continuous variables in the baseline table are expressed by the median (P25, P75), and the categorical variables are expressed by the number of use cases n and percentage. For categorical variables, Chi-square tests were used to determine. For continuous variables, the Spearman rank correlation coefficient was used to analyze the difference between the age groups.

### 3.2 The relationship between B-vitamins intake and hypertension in weighted logistics analysis

In the weighted logistics regression analysis, we take P25, P50 and P75 as the dividing points according to dietary B-vitamins intake, divide continuous variables into four equal parts, and the data are represented by mean and value range of the original data (Table 2).

**Table 2:** Intake range of B vitamins at quartile.

	Continuous	Q1	Q2	Q3	Q4
Vitamin B1(mg/day)	4.512 (0.048-253.955)	0.811 (0.048-1.121)	1.376 (1.122-1.667)	2.073 (1.668-2.581)	13.558 (2.584-253.955)
Vitamin B2(mg/day)	3.71 (0.073-104.935)	0.991 (0.073-1.375)	1.691 (1.376-2.028)	2.544 (2.029-3.17)	9.508 (3.17-104.935)
Niacin (mg/day)	34.342 (1.102-1547.048)	12.888 (1.102-18.175)	22.215 (18.192-26.406)	32.022 (26.414-38.956)	69.391 (38.98-1547.048)
Vitamin B6 (mg/day)	4.899 (0.091-503.114)	0.978 (0.091-1.395)	1.78 (1.395-2.199)	2.941 (2.201-3.938)	13.906 (3.943-503.114)
Folate (mcg/day)	717.885 (0-13056)	205.845 (1-312)	417.998 (312-539)	740.973 (540-979)	1508.298 (979-13056)
Choline (mg/day)	327.15 (22.6-1806)	149.41 (22.6-206.7)	251.051 (207.1-294.95)	348.567 (294.95-410.8)	553.659 (410.9-1806)
Vitamin B12 (mcg**/**day)	125.572 (0.07-12620.66)	1.616 (0.07-2.685)	3.799 (2.685-5.21)	8.172 (5.22-13.19)	485.709 (13.21-12620.66)

After adjusting for covariates such as demographic, questionnaire, laboratory, and the other meals in the dietary survey data, vitamin B1 was negatively associated with hypertension in ≥60 years old, Q3:Q1 OR (95% CI) = 0.22 (0.05, 0.91), Q4:Q1 OR (95% CI) = 0.27 (0.08, 0.91). Vitamin B2 shown a negative association with hypertension in the total population, and its Q4: Q1 OR (95%CI) = 0.39 (0.15, 0.99), this association was also found in people 40–59 years old, Q3: Q1 OR (95%CI) = 0.27 (0.09, 0.81) and Q4: Q1 OR (95%CI) = 0.16 (0.04, 0.66), and in people ≥60 years old, Q3: Q1 OR (95%CI) = 0.25 (0.07, 0.84). A negative correlation trend was also found between vitamin B2 continuous variable and hypertension in aged 40–59 years, OR (95%CI) = 0.52 (0.33, 0.83). Niacin was positively correlated with the population 40–59 years old, Q3:Q1 OR (95% CI) = 3.78 (1.22, 11.71). Vitamin B6 was negatively associated with hypertension in people ≥60 years old, Q3:Q1 OR (95%CI) = 0.29 (0.09, 0.92). Choline was negatively associated with the population ≥60 years old, Q4: Q1 OR (95%CI) = 0.25 (0.07, 0.93), and a negative correlation trend was also found between them, OR (95%CI) = 0.73 (0.56, 0.95). Vitamin B12 has also been found to have a positive association with hypertension in total population Q3: Q1 OR (95%CI) = 3.12 (1.78, 5.48), Q4: Q1 OR (95%CI) = 2.26 (1.15, 4.44), and in people 20–39 years old, Q3: Q1 OR (95%CI) = 12.28 (3.54, 42.58). A positive trend was also found between vitamin B12 continuous variable and hypertension in total people and 20–39 years old, OR (95%CI) = 1.39 (1.13, 1.71), 1.82 (1.23, 2.69). (Table 3).

**Table 3:** Weighted logistics regression analysis between B vitamins intake and hypertension in NHANES adults.

Variable	Hypertension OR (95%CI)				
	Continuous	Q1	Q2	Q3	Q4
**Total population**					
Vitamin B1(mg/day)	0.86 (0.67, 1.11)	ref.	0.70 (0.31, 1.56)	0.72 (0.30, 1.75)	0.58 (0.25, 1.36)
Vitamin B2(mg/day)	0.74 (0.56, 1.01)	ref.	0.71 (0.44, 1.16)	0.64 (0.38, 1.08)	0.39 (0.15, 0.99)*
Niacin(mg/day)	1.02 (0.79, 1.32)	ref.	1.50 (0.77, 2.93)	1.40 (0.75, 2.61)	1.28 (0.52, 3.10)
Vitamin B6(mg/day)	0.98 (0.77, 1.24)	ref.	1.69 (0.85, 3.35)	1.63 (0.89, 2.98)	1.17 (0.51, 2.65)
Folate (mcg/day)	1.11 (0.86, 1.43)	ref.	1.02 (0.50, 2.09)	0.94 (0.52, 1.68)	1.36 (0.61, 3.01)
Choline (mg/day)	1.30 (1.08, 1.58)*	ref.	1.00 (0.39, 2.61)	1.51 (0.74, 3.10)	1.96 (0.84, 4.58)
Vitamin B12 (mcg/day)	1.39 (1.13, 1.71)*	ref.	0.99 (0.46, 2.11)	3.12 (1.78, 5.48)*	2.26 (1.15, 4.44)*
**20-39 years old**					
Vitamin B1(mg/day)	0.92 (0.54, 1.56)	ref.	0.65 (0.18, 2.32)	0.78 (0.14, 4.43)	0.67 (0.13, 3.53)
Vitamin B2(mg/day)	0.83 (0.56, 1.22)	ref.	0.89 (0.38, 2.06)	1.74 (0.64, 4.71)	0.29 (0.06, 1.45)
Niacin(mg/day)	1.47 (0.99, 2.15)	ref.	2.14 (0.44, 10.34)	2.79 (0.66, 11.80)	3.87 (0.77, 19.51)
Vitamin B6(mg/day)	1.38 (0.86, 2.23)	ref.	2.99 (0.91, 9.77)	7.68 (1.67, 35.34)	2.46 (0.48, 12.61)
Folate (mcg/day)	1.26 (0.78, 2.03)	ref.	1.02 (0.40, 2.60)	1.57 (0.49, 5.04)	1.79 (0.41, 7.92)
Choline (mg/day)	1.49 (0.83, 2.68)	ref.	0.78 (0.15, 4.11)	1.64 (0.49, 5.56)	2.43 (0.38, 15.58)
Vitamin B12 (mcg/day)	1.82 (1.23, 2.69)*	ref.	1.62 (0.57, 4.59)	12.28 (3.54, 42.58)*	3.32 (0.68, 16.12)
**40-59 years old**					
Vitamin B1(mg/day)	0.89 (0.55, 1.46)	ref.	0.74 (0.23, 2.40)	0.70 (0.20, 2.45)	0.65 (0.13, 3.17)
Vitamin B2(mg/day)	0.52 (0.33, 0.83)*	ref.	0.68 (0.27, 1.76)	0.27 (0.09, 0.81)*	0.16 (0.04, 0.66)*
Niacin(mg/day)	1.01 (0.69, 1.48)	ref.	3.33 (1.01, 11.02)*	3.78 (1.22, 11.71)*	1.93 (0.59, 6.34)
Vitamin B6(mg/day)	0.98 (0.65, 1.49)	ref.	2.25 (0.59, 8.65)	1.91 (0.51, 7.15)	1.35 (0.32, 5.77)
Folate (mcg/day)	0.82 (0.53, 1.26)	ref.	0.52 (0.18, 1.54)	0.28 (0.06, 1.23)	0.47 (0.13, 1.72)
Choline (mg/day)	1.50 (0.98, 2.19)	ref.	1.14 (0.48, 2.74)	2.46 (0.80, 7.53)	2.74 (0.71, 10.50)
Vitamin B12 (mcg/day)	1.16 (0.81, 1.64)	ref.	0.82 (0.30, 2.25)	1.72 (0.46, 6.50)	1.35 (0.45, 4.02)
**≥60 years old**					
Vitamin B1(mg/day)	0.68 (0.46, 1.02)	ref.	0.43 (0.12, 1.49)	0.22 (0.05, 0.91)*	0.27 (0.08, 0.91)*
Vitamin B2(mg/day)	0.75 (0.49, 1.15)	ref.	0.31 (0.09, 1.12)	0.25 (0.07, 0.84)*	0.32 (0.09, 1.21)
Niacin(mg/day)	0.76 (0.49, 1.18)	ref.	0.69 (0.14, 3.39)	0.34 (0.09, 1.27)	0.44 (0.10, 1.98)
Vitamin B6(mg/day)	0.81 (0.54, 1.21)	ref.	1.11 (0.29, 4.20)	0.29 (0.09, 0.92)*	0.66 (0.18, 2.44)
Folate (mcg/day)	1.33 (0.87, 2.05)	ref.	1.60 (0.60, 4.26)	1.16 (0.38, 3.48)	2.63 (0.72, 9.59)
Choline (mg/day)	0.73 (0.56, 0.95)*	ref.	0.37 (0.10, 1.35)	0.55 (0.22, 1.38)	0.25 (0.07, 0.93)*
Vitamin B12 (mcg/day)	1.63 (1.04, 2.54)*	ref.	0.63 (0.20, 1.99)	1.61 (0.46, 5.63)	2.85 (0.76, 10.69)

Note:

1.* Statistical significance, P < 0.05.

2. The weighted logistic regression model was used to analyze the association between B vitamins and hypertension.All analyses were adjusted for age group, alcohol consumption, BMI, education level, race, marital status, serum cotinine, PIR, gender, exercise, diabetes status, CVD, kidney disease, taking anti-hypertensive drugs, energy intake, fat intake, protein intake, sodium intake, potassium intake, and carbohydrate intake.

### 3.3 The relationship between B vitamins intake and systolic pressure in weighted linear regression analysis

After adjusting for all covariates (including taking anti-hypertensive drugs), we found no association between the 7 studied B vitamins, either continuous or categorical, and systolic pressure in the total population (P > 0.05). There was a negative association between vitamin B1 and systolic pressure in the population 20–39 years old and ≥60 years old. The β (95%CI) of Q2:Q1 and Q3:Q1 in 20–39 years old were −3.76 (−5.95,-1.57), −5.21 (−8.14, −2.28) respectively, and in ≥60 years old, Q2:Q1 β (95%CI) =−7.71 (−14.08, −1.34). Vitamin B12 was inconsistently positive associated with systolic pressure between 20–39 years old, Q2:Q1 and Q3:Q1 β (95%CI) =2.94 (0.26, 5.62), 5.03 (2.55, 7.50) respectively (Table 4).

**Table 4:** Weighted linear regression analysis between B vitamins intake and systolic pressure in NHANES adults.

Variable		Systolic pressure β (95%CI)			
	Continuous	Q1	Q2	Q3	Q4
**Total population**					
Vitamin B1(mg/day)	−0.68 (−1.78, 0.42)	ref.	−2.80 (−6.77, 1.18)	−1.73 (−4.88, 1.41)	−3.11 (−6.86, 0.63)
Vitamin B2(mg/day)	−0.80 (−1.83, 0.22)	ref.	−1.13 (−4.34, 2.08)	−0.60 (−3.67, 2.48)	−2.71 (−6.04, 0.62)
Niacin(mg/day)	−0.22 (−1.13, 0.69)	ref.	0.43 (−3.11, 3.96)	1.13 (−2.23, 4.50)	−0.62 (−3.90, 2.67)
Vitamin B6(mg/day)	0.15 (−0.77, 1.06)	ref.	0.69 (−3.62, 5.01)	0.80 (−3.70, 5.31)	0.63 (−2.69, 3.96)
Folate (mcg/day)	0.49 (−0.77, 1.75)	ref.	1.67 (−0.90, 4.23)	2.06 (−0.84, 4.95)	1.82 (−2.49, 6.12)
Choline (mg/day)	−0.48 (−1.33, 0.38)	ref.	−2.03 (−5.06, 1.00)	−2.76 (−5.51, 0.01)	−1.62 (−4.55, 1.30)
Vitamin B12 (mcg/day)	0.40 (−0.85, 1.65)	ref.	−0.13 (−2.41, 2.14)	1.94 (−1.11, 4.99)	0.73 (−2.71, 4.18)
**20-39 years old**					
Vitamin B1(mg/day)	−0.67 (−1.69, 0.35)	ref.	−3.76 (−5.95, −1.57)*	−5.21 (−8.14, −2.28)*	−2.63 (−5.53, 0.27)
Vitamin B2(mg/day)	−0.61 (−1.59, 0.38)	ref.	−2.49 (−5.85, 0.87)	−1.87 (−4.48, 0.73)	−2.64 (−6.09, 0.81)
Niacin(mg/day)	0.51 (−0.44, 1.45)	ref.	1.00 (−1.69, 3.69)	1.23 (−3.18, 5.65)	1.75 (−0.97, 4.46)
Vitamin B6(mg/day)	−0.07 (−1.26, 1.13)	ref.	0.70 (−3.36, 4.76)	2.22 (−3.42, 7.86)	−0.40 (−4.27, 3.47)
Folate (mcg/day)	−0.01 (−1.31, 1.29)	ref.	−2.23 (−4.55, 0.08)	−1.21 (−4.29, 1.86)	−0.70 (−4.48, 3.09)
Choline (mg/day)	−0.32 (−1.76, 1.12)	ref.	−2.42 (−5.81, 0.97)	−1.75 (−5.39, 1.89)	−1.82 (−6.32, 2.68)
Vitamin B12 (mcg/day)	0.49 (−0.39, 1.36)	ref.	2.94 (0.26, 5.62)*	5.03 (2.55, 7.50)*	0.19 (−2.10, 2.48)
**40-59 years old**					
Vitamin B1(mg/day)	−0.05 (−2.13, 2.03)	ref.	2.47 (−3.07, 8.01)	7.21 (−0.35, 14.76)	0.35 (−6.70, 7.40)
Vitamin B2(mg/day)	−1.56 (−3.18, 0.07)	ref.	2.19 (−4.41, 8.80)	2.99 (−2.81, 8.78)	−3.27 (−8.92, 2.39)
Niacin(mg/day)	−0.53 (−2.83, 1.77)	ref.	3.56 (−2.40, 9.52)	6.17 (0.83, 11.52)	−0.37 (−8.36, 7.62)
Vitamin B6(mg/day)	0.68 (−0.95, 2.30)	ref.	3.58 (−0.83, 7.99)	5.45 (−1.91, 8.98)	2.85 (−2.54, 8.24)
Folate (mcg/day)	0.68 (−0.83, 2.19)	ref.	4.98 (−0.48, 10.44)	6.39 (−1.16, 13.94)	3.75 (−1.81, 9.31)
Choline (mg/day)	−1.14 (−2.99, 0.71)	ref.	−1.54 (−4.84, 1.77)	−3.77 (−8.41, 0.87)	−3.13 (−8.94, 2.69)
Vitamin B12 (mcg/day)	−0.09 (−1.52, 1.33)	ref.	0.87 (−3.15, 4.89)	0.60 (−3.94, 5.13)	0.06 (−4.65, 4.77)
**≥60 years old**					
Vitamin B1(mg/day)	−0.97 (−3.20, 1.26)	ref.	−7.71 (−14.08, −1.34)*	−6.29 (−12.66, 0.09)	−5.83 (−13.00, 1.35)
Vitamin B2(mg/day)	−0.29 (−2.04, 1.46)	ref.	−3.71 (−7.99, 0.56)	−2.68 (−7.24, 1.87)	−2.04 (−7.85, 3.77)
Niacin(mg/day)	−0.90 (−2.74, 0.94)	ref.	−3.19 (−10.75, 4.37)	−3.48 (−9.72, 2.76)	−3.44 (−10.57, 3.68)
Vitamin B6(mg/day)	−0.25 (−1.90, 1.39)	ref.	−1.04 (−9.97, 7.88)	−3.46 (−10.76, 3.83)	−0.71 (−7.37, 5.94)
Folate (mcg/day)	0.67 (−1.89, 3.23)	ref.	3.32 (−3.67, 10.31)	2.70 (−2.98, 8.38)	2.85 (−5.85, 11.55)
Choline (mg/day)	−0.41 (−3.04, 2.22)	ref.	−5.52 (−13.35, 2.30)	−3.70 (−11.99, 4.58)	−2.28 (−11.08, 6.52)
Vitamin B12 (mcg/day)	−0.08 (−2.24, 2.09)	ref.	−8.78 (−13.47, −4.08)	−4.88 (−11.09, 1.34)	−3.92 (−10.31, 2.46)

Note:

1.* Statistical significance, P < 0.05.

2. The weighted linear regression model is used to analyze the association between B vitamins and systolic pressure. All analyses were adjusted for age group, alcohol consumption, BMI, education level, race, marital status, serum cotinine, PIR, gender, exercise, diabetes status, CVD, kidney disease, taking anti-hypertensive drugs, energy intake, fat intake, protein intake, sodium intake, potassium intake, and carbohydrate intake.

### 3.4 The relationship between B vitamins intake and diastolic pressure in weighted linear regression analysis

Table 5 shown that the association between dietary B-vitamins intake and blood pressure was more closely related to diastolic pressure. After adjusting for all covariates, the negative association between vitamin B1 and diastolic pressure had a linear relationship in the total population and in people ≥60 years old, β (95% CI) were −0.89 (−1.44, −0.35), −1.50 (−2.57, −0.43), respectively. In addition, when the four categories were compared, there was a negative correlation between vitamin B1 and the diastolic pressure of the total population, and compared with Q1, Q2-Q4 β (95%CI) were −2.90(−4.65, −1.15), −3.88(−5.69, −2.08) and −3.37(−5.22, −1.53), respectively. There was a negative association between vitamin B1 and diastolic pressure in ≥60 years old, and compared with Q1, Q3-Q4 β (95%CI) were −5.66 (−9.01, −2.31) and −5.10(−9.32, −0.87), respectively. The linear association between vitamin B2 (continuous variable) and diastolic pressure was β (95%CI) = −1.40 (−1.96, −0.84) for the total population, β (95%CI) = −1.86 (−3.24, −0.47) for 40−59 years old and β (95%CI) = −1.93 (−2.94, −0.91) for ≥60 years old. For the quartile variables of vitamin B2 and diastolic pressure Q2 - Q4 β (95% CI) were −2.40 (−3.65, −1.14), −3.12 (−5.36, −0.89) and −4.64 (−6.52, −2.76) respectively in total population and Q4:Q1 β (95% CI) was −5.69 (−9.40, −1.98). In the ≥ 60 years old, the negative association between vitamin B2 and diastolic pressure Q2 - Q4 β(95% CI) were −4.15 (−7.54, −0.75),−6.22 (−10.13,-2.30) and −6.39(−9.36, −3.42), respectively.

**Table 5:** Weighted linear regression analysis between B vitamins intake and diastolic pressure in NHANES adults.

Variable		Diastolic pressure β (95%CI)			
	Continuous	Q1	Q2	Q3	Q4
**Total population**					
Vitamin B1(mg/day)	−0.89 (−1.44, −0.35)*	ref.	−2.90 (−4.65, −1.15)*	−3.88 (−5.69, −2.08)*	−3.37 (−5.22, −1.53)*
Vitamin B2(mg/day)	−1.40 (−1.96, −0.84)*	ref.	−2.40 (−3.65, −1.14)*	−3.12 (−5.36, −0.89)*	−4.64 (−6.52, −2.76)*
Niacin(mg/day)	−0.88 (−1.39, −0.38)*	ref.	−0.22 (−1.82, 1.39)	−2.08 (−4.29, 0.14)	−2.24 (−3.99, −0.49)*
Vitamin B6(mg/day)	−0.73 (−1.21, −0.24)*	ref.	0.25 (−2.51, 3.01)	−0.58 (−3.49, 2.32)	−1.83 (−3.75, 0.09)
Folate (mcg/day)	−0.59 (−1.11, −0.07)*	ref.	−2.65 (−4.73, −0.57)*	−1.07 (−2.78, 0.65)	−2.75 (−4.48, −1.03)*
Choline (mg/day)	−0.31 (−1.12, 0.51)	ref.	0.20 (−1.73, 2.13)	−0.82 (−3.23, 1.60)	−0.55 (−3.25, 2.14)
Vitamin B12 (mcg/day)	−0.08 (−0.57, 0.40)	ref.	−0.44 (−2.65, 1.77)	0.12 (−2.33, 2.56)	−0.46 (−1.87, 0.94)
**20-39 years old**					
Vitamin B1(mg/day)	−0.64 (−1.51, 0.24)	ref.	−2.48 (−5.26, 0.30)	−3.24 (−6.77, 0.28)	−2.37 (−5.05, 0.31)
Vitamin B2(mg/day)	−0.87 (−1.84, 0.11)	ref.	−1.11 (−3.99, 1.77)	−1.51 (−4.05, 1.03)	−2.80 (−6.39, 0.80)
Niacin(mg/day)	−0.46 (−1.53, 0.60)	ref.	2.36 (−1.00, 5.73)	−1.04 (−5.00, 2.92)	0.17 (−3.42, 3.76)
Vitamin B6(mg/day)	−0.68 (−1.76, 0.40)	ref.	1.62 (−1.99, 5.22)	0.31 (−3.42, 4.04)	−1.09 (−4.53, 2.35)
Folate (mcg/day)	−0.67 (−1.51, 0.17)	ref.	−2.46 (−4.87, −0.06)	−0.72 (−3.04, 1.60)	−3.21 (−6.01, −0.41)*
Choline (mg/day)	−0.30 (−1.20, 0.61)	ref.	−1.77 (−4.90, 1.35)	−0.24 (−3.08, 2.61)	−1.87 (−4.99, 1.25)
Vitamin B12 (mcg/day)	0.42 (−0.64, 1.49)	ref.	1.88 (−0.58, 4.34)	2.33 (−0.53, 5.20)	1.05 (−2.08, 4.17)
**40-59 years old**					
Vitamin B1(mg/day)	−0.45 (−1.80, 0.91)	ref.	−2.36 (−5.33, 0.61)	−2.28 (−6.80, 2.23)	−2.22 (−6.56, 2.12)
Vitamin B2(mg/day)	−1.86 (−3.24, −0.47)*	ref.	−1.84 (−5.03, 1.35)	−1.87 (−5.65, 1.91)	−5.69 (−9.40, −1.98)*
Niacin(mg/day)	−0.53 (−1.54, 0.47)	ref.	1.02 (−1.76, 3.81)	1.32 (−3.23, 5.88)	−1.08 (−4.13, 1.97)
Vitamin B6(mg/day)	0.18 (−0.70, 1.07)	ref.	1.36 (−1.14, 3.86)	1.07 (−2.62, 4.76)	1.03 (−1.57, 3.62)
Folate (mcg/day)	−0.09 (−0.95, 0.78)	ref.	−1.12 (−5.18, 2.95)	0.20 (−3.37, 3.78)	−0.79 (−4.09, 2.51)
Choline (mg/day)	−1.23 (−2.63, 0.16)	ref.	1.94 (−1.04, 4.92)	−1.13 (−4.32, 2.07)	−2.19 (−6.90, 2.52)
Vitamin B12 (mcg/day)	0.26 (−0.87, 1.39)	ref.	0.63 (−3.85, 5.12)	0.75 (−3.96, 5.47)	0.92 (−3.07, 4.92)
**≥60 years old**					
Vitamin B1(mg/day)	−1.50 (−2.57, −0.43)*	ref.	−3.49 (−7.43, 0.45)	−5.66 (−9.01, −2.31)*	−5.10 (−9.32, −0.87)*
Vitamin B2(mg/day)	−1.93 (−2.94, −0.91)*	ref.	−4.15 (−7.54, −0.75)*	−6.22 (−10.13, −2.30)*	−6.39 (−9.36, −3.42)*
Niacin(mg/day)	−1.69 (−3.10, −0.27)*	ref.	−4.87 (−8.23, −1.50)*	−5.83 (−10.29, −1.37)*	−6.07 (−10.82, −1.32)*
Vitamin B6(mg/day)	−1.89 (−3.02, −0.75)*	ref.	−2.03 (−6.30, 2.23)	−3.00 (−7.08, 1.08)	−5.82 (−9.99, −1.66)*
Folate (mcg/day)	−0.90 (−1.79, −0.01)*	ref.	−3.56 (−7.06, −0.06)*	−1.07 (−4.10, 1.97)	−3.92 (−6.97, −0.87)*
Choline (mg/day)	0.12 (−1.51, 1.74)	ref.	−0.81 (−3.99, 2.38)	−1.59 (−6.12, 2.95)	0.76 (−4.24, 5.75)
Vitamin B12 (mcg/day)	−1.40 (−2.18, −0.63)*	ref.	−6.27 (−9.61, −2.93)*	−6.23 (−10.02, −2.45)*	−6.26 (−8.97, −3.54)*

Note:

1.* Statistical significance, P < 0.05.

2. The weighted linear regression model is used to analyze the association between B vitamins and diastolic pressure. All analyses were adjusted for age group, alcohol consumption, BMI, education level, race, marital status, serum cotinine, PIR, gender, exercise, diabetes status, CVD, kidney disease, taking anti-hypertensive drugs, energy intake, fat intake, protein intake, sodium intake, potassium intake, and carbohydrate intake.

Niacin shown a negative linear relationship with the diastolic pressure of the total population, whose continuous variable β (95%CI) =−0.88 (−1.39, −0.38), and this association was also reflected in Q4: Q1 in the quartile variables, β (95% CI) =−2.24 (−3.99, −0.49). While for ≥60 years old, the association between niacin and diastolic pressure was reflected in the quartile variables and continuous variable, Q2-Q4: Q1 β (95% CI) = −4.87(−8.23, −1.50), −5.83(−10.29, −1.37) and −6.07(−10.82, −1.32), respectively, and continuous variable β (95%CI) =−1.69 (−3.10, −0.27)

Vitamin B6 was negatively associated with total population diastolic pressure β (95% CI) = −0.73 (−1.21, −0.24). And in ≥60 years old, this connection still exists β (95%CI) = −1.89 (−3.02, −0.75), Q4:Q1 β (95%CI) = −5.82 (−9.99, −1.66).

In the total population, the negative correlation between folate and diastolic pressure appeared in the continuous variable β (95%CI) = −0.59 (−1.11, −0.07) and the quartile variables Q2:Q1 β (95%CI) = −2.65 (−4.73, −0.57), Q4: Q1 β (95%CI) = −2.75 (−4.48, −1.03). This association also existed in≥60 years old, whose continuous variable β (95%CI)= −0.90 (−1.79,-0.01) and Q2:Q1 β (95%CI) = −3.56 (−7.06, −0.06), Q4: Q1β (95%CI) = −3.92 (−6.97, −0.87). However, in the 20−39 years old, the negative association between folate and diastolic pressure was only reflected in Q4: Q1 β (95%CI) = −3.21 (−6.01, −0.41).

In the ≥ 60 years old, there was a negative correlation between vitamin B12 and diastolic pressure β (95%CI) = −1.40 (−2.18, −0.63). In comparison with Q1, the Q2-Q4β (95%CI) was −6.27 (−9.61, −2.93), −6.23 (−10.02, −2.45) and −6.26 (−8.97, −3.54), respectively.

### 3.5 The relationship between B vitamins intake and hypertension by weighted RCS model

**Fig 2:**
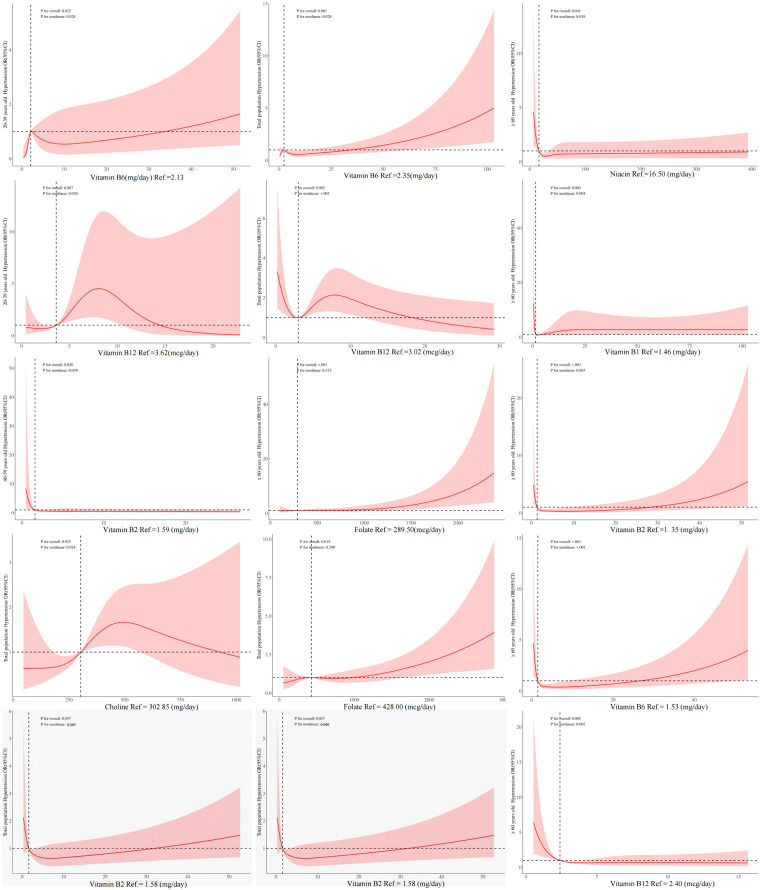
The linear/ nonlinear relationship between B vitamins intake and hypertension by weighted RCS model. Note: 1. Statistical significance, P < 0.05. 2.The red solid line represents the OR value, the red shadow represents the OR (95% CI), the horizontal dashed line represents the OR reference value = 1, and the vertical dashed line represents the value corresponding to B vitamins when OR = 1.

For hypertension, different types of B vitamins intake have different linear or non-linear association in different age group. Vitamin B2 has a nonlinear “L” type association with hypertension in more than 40 years old and total population, and vitamin B1 and niacin has a nonlinear reverse “L” type association with hypertension in people ≥60 years old. For the total population, vitamin B6, choline and vitamin B12, all had a nonlinear association with hypertension. The nonlinear association between vitamin B6 and vitamin B12 and hypertension also exists in the population aged 20–39 years, and the association direction was consistent with the total population. Above all *P* for overall and *P* for nonlinear were <0.05. For the total population and people ≥60 years old there were a linear association between folate and hypertension, *P* for overall<0.05, *P* for nonlinear>0.05 (Fig 2). However, there were no linear and non-linear associations between other unmentioned vitamins and hypertension in different populations (S1 Fig).

### 3.6 The relationship between B vitamins intake and systolic pressure by weighted RCS model

**Fig 3:**
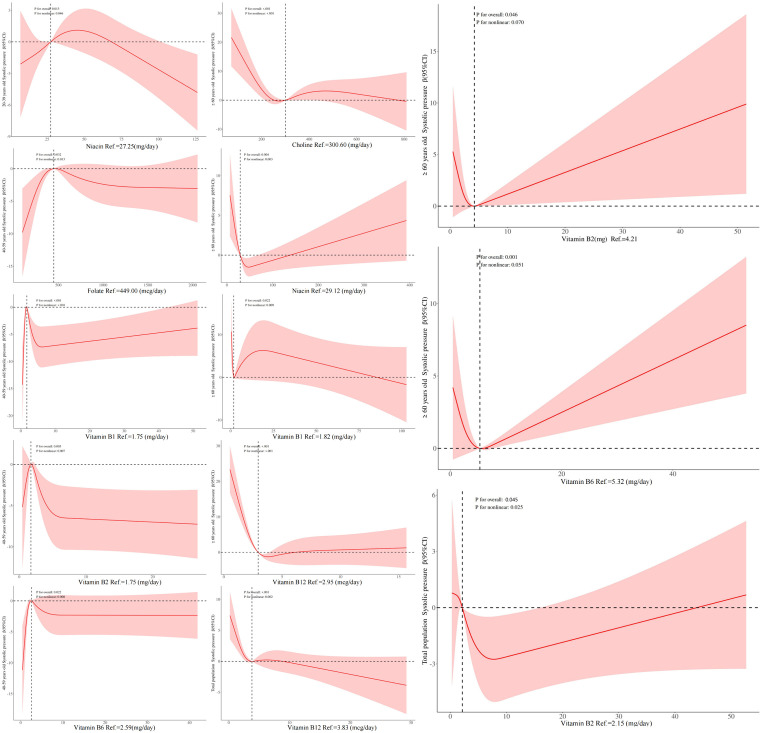
The linear/nonlinear relationship between B vitamins intake and systolic pressure by weighted RCS model. Note: 1. Statistical significance, P < 0.05. 2.The red solid line represents the β value, the red shadow represents the β (95% CI), the horizontal dashed line represents the β reference value = 0, and the vertical dashed line represents the value corresponding to B vitamins when β = 1.

The associations between vitamin B1, vitamin B2, vitamin B6, folate and systolic pressure were consistent with a nonlinear relationship of increasing and then decreasing in 40–59 years old, and in people ≥60 years old. There was an increase and then decrease trend between vitamin B1 and systolic pressure, whereas the trend between choline and systolic pressure was for systolic pressure to decrease and then level off as choline increased, *P* for overall<0.05, *P* for nonlinear<0.05. In total population, vitamin B2 was linearly and positively associated with systolic pressure, and this association was similarly found in ≥60 years old, and in≥60 years old we also found vitamin B6 to be linearly and positively associated with systolic pressure *P* for overall<0.05, *P* for nonlinear >0.05. Trends in the association between niacin and systolic pressure differed between 20–39 years old (younger) and≥ 60 years old (older), with an inverse “U” shaped association in younger and a positive “U” shaped association in older. The nonlinear association between vitamin B12 and systolic pressure was of the “L” type in the total population and ≥60 years old (Fig 3). However, there were no linear and non-linear associations between other unmentioned vitamins and systolic pressure in different populations (S2 Fig).

### 3.7 The relationship between B vitamins intake and diastolic pressure by weighted RCS model

**Fig 4:**
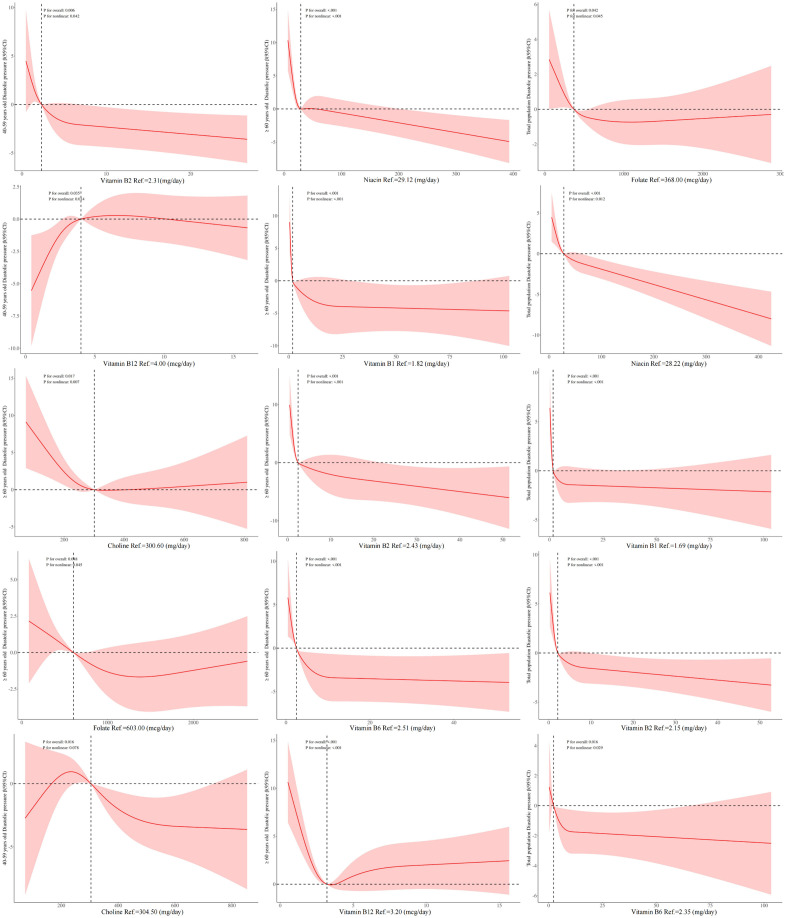
The linear/nonlinear relationship between B vitamins intake and diastolic pressure by weighted RCS model. Note: 1. Statistical significance, P < 0.05. 2.The red solid line represents the β value, the red shadow represents the β (95% CI), the horizontal dashed line represents the β reference value = 0, and the vertical dashed line represents the value corresponding to B vitamins when β = 1.

In the total population and ≥60 years old, there were an “L” -shaped nonlinear association between vitamin B1, vitamin B2, vitamin B6, folate, niacin and diastolic pressure. This association between vitamin B2 and diastolic pressure has also been found in 40–59 years old. The RCS results shown that the association trend of vitamin B12 with diastolic pressure was inconsistent in different age groups. In 40–59 years old, it was an inverted “L” shape, while in the ≥ 60 years old, it was an “L” shape. There was a nonlinear L-shaped association between choline and diastolic pressure in ≥60 years old, while in 40–59 years old, when the intake was greater than 200 mg/day, it shown a linear negative correlation with diastolic pressure (Fig 4). However, there were no linear and non-linear associations between other unmentioned vitamins and diastolic pressure in different populations (S3 Fig).

## 4. Discussion

Our study revealed that dietary intake of B vitamins exhibits complex, age-dependent associations with blood pressure, with several clinically relevant patterns emerging from the data. Notably, we observed that vitamins B1 and B2 consistently demonstrated protective associations against hypertension, particularly in adults aged 60 years and older. A novel and significant finding was the identification of a positive correlation between excessive choline intake and hypertension in the general population—a relationship that warrants further investigation. Furthermore, the application of restricted cubic spline models uncovered important non-linear dose-response relationships that were not captured by conventional linear regression, highlighting nuanced thresholds for vitamin B12 and paradoxical associations for vitamins B2 and B6 with diastolic pressure. These findings carry direct implications for dietary guidance, especially for older adults, among whom hypertension prevalence was highest and B vitamin intake was significantly elevated, likely reflecting intentional supplementation to address nutritional adequacy. The results collectively suggest that tailored nutritional strategies, rather than uniform recommendations, may be necessary to optimize cardiovascular health across different age groups.

In our study, vitamin B1, B2, niacin, B6 and folate were highly intake in the total population and people age ≥ 60 years old. There were negative association among vitamin B1 intake, hypertension, systolic and diastolic pressure in RCS, and this association disappeared when it reached the inflection point ≈1.46 (mg/day). Existing study have shown that the recommended intake of vitamin B1 for women and men was 1.1 and 1.2 mg/day [37,38] and the inflection point of 1.46 was greater than the recommended intake, which indicates that for blood pressure control, excessive intake of vitamin B1 may be helpful for stabilizing blood pressure. Previous report had indicated that a large intake of vitamin B1 can reverse the risk of hypertension [18]. This might be because vitamin B1 is a potential low-cost and effective intervention method. The vitamin B1 analogue, pyridoxal phosphate, prevents diabetic-induced diastolic dysfunction and heart failure through the survival pathway mediated by Akt/Pim-1 [39,40]. Moreover, thiamine, mediated by glucose and insulin, has a protective effect on the proliferation of human lower arterial smooth muscle cells [41], which is crucial in the formation of atherosclerotic plaques [38,42], and we believe that this phenomenon may also exist in the general population.

The RCS results indicated that the effects of vitamin B2 and B6 on blood pressure in the elderly were L-shaped. With the increase of intake, blood pressure gradually decreased. The inflection points were roughly distributed at vitamin B2 ≈ 1.35 (mg/day) and vitamin B6 ≈ 1.53 (mg/day), and there was no difference in the effect after the inflection point. These findings may be explained through known mechanisms of B vitamins. Existing study have shown that the recommended intake of vitamin B2 for women and men are 1.1 and 1.3 mg/day [43]. Importantly, previous research has demonstrated that vitamin B2 can regenerate glutathione (GSH) through FAD-dependent glutathione reductase, neutralize reactive oxygen species (ROS), promote the release of NO, and dilate blood vessels [44,45]. Similarly, vitamin B6 acts as a PLP-assisted antioxidant enzyme (such as superoxide dismutase), reduces the generation of lipid peroxidation products (such as MDA) [46], and promotes H₂S production through PLP-dependent cystathionine β -synthase (CBS), and cooperates with NO to dilate blood vessels [47,48]. Notably, our observed inflection points align with these mechanisms, as the synergistic effect of the two, B2 enhances the synthesis of GSH, and B6 reduces the generation of ROS. The combination of the two can reduce oxidative stress markers by more than 30% [44,48]. Our results suggest these known biological mechanisms may reach their maximum functional thresholds at these intake levels.

Our research results shown that there were a “U” -shaped nonlinear relationship between niacin and hypertension and systolic pressure in the population 20−39 years old, with an inflection point of approximately 16.50 mg/day (the recommended intake of 16 for men and 14 for women (mg/ day) [49]). However, in ≥60 years old, there was an L-shaped nonlinear relationship and the inflection point was approximately 28 mg/day which was much higher than the recommended intake. This age-specific contradictory effect may be related to the bidirectional dose-dependent mechanism of niacin: Low-dose niacin mediates vasodilation and anti-inflammatory effects by activating the GPR109A receptor [50,51], while high-dose niacin aggravates vascular damage by promoting inflammatory pathways and oxidative stress [52,53]. It was reported that low-dose niacin was an effective lipid regulator, which can inhibit the lipopolification of adipocytes, reduce the release of free fatty acids, improve insulin sensitivity and endothelial function, and lower the risk of cardiovascular diseases [54]. However, at high doses, the metabolite of niacin, N1-methyl-4-pyridine-3-formamide (4PY), accumulates significantly. 4PY activates the NLRP3 inflammasome [52], promotes the secretion of IL-1β and IL-18, induces vascular endothelial inflammation and arteriosclerosis [55], and by inhibiting the activity of SIRT1 deacetylase, it leads to the dysfunction of endothelial nitric oxide synthase (eNOS) [56]. The activity of niacin metabolic enzyme ACMSD was higher in the young population, and it was more likely to generate the pro-inflammatory end product 4PY [57]. However, in the elderly, the expression of GPR109A receptor was down-regulated, and the sensitivity to the vasodilatory effect of niacin is reduced [58].

In our study, it was found that choline was positively associated with hypertension in the total population, and this association was highlighted when the intake exceeded 302.85(mg/day). For 40–59 years old, there was a negative nonlinear association between choline and diastolic pressure, with an inflection point of approximately 304.5 (mg/day). For ≥60 years old, whether it was hypertension, diastolic pressure or systolic pressure, they all shown a negative nonlinear correlation inflection point≈300.6 (mg/day), and the association difference were not obvious after the inflection point. A cross-sectional study based on NHANES found an inverted J association between choline intake and ASCVD [59]. An appropriate amount of choline improves lipid metabolism by promoting the synthesis of phosphatidylcholine and inhibits liver steatosis, thereby reducing peripheral vascular resistance [60]. However, excessive intake may intensify oxidative stress in the liver and counteract its benefits [61]. The decreased choline absorption rate and the reduced diversity of intestinal flora in the elderly may lead to a decrease in the production of TMAO, and supplementation of choline in the elderly can significantly increase plasma choline concentration and improve vascular endothelial function [62]. Therefore, the association between choline and blood pressure varies among people of different age groups.

Our research found that there was a nonlinear inverted U-shaped association between vitamin B12 and hypertension in the total population and 20−39 years old. It shown a positive correlation when the intake exceeds 3.0 mcg/day (3.62 mcg/day for 20−39 years old), but a negative nonlinear association in the population aged ≥60 years old, with an inflection point = 2.40 mcg/day. This association was also reflected in the systolic pressure of ≥60 years old and total population, with inflection points of 2.95 and 3.83 mcg/day respectively. Folate shown a linear positive correlation with hypertension in≥60 years old when the intake exceeds 500 mcg/day, which was consistent with the result found in the total population. For people 40−59 years old, there was a nonlinear inverted U-shaped association between folate and systolic pressure, but β < 0, which was consistent with the association between vitamin B12 and diastolic pressure. For diastolic pressure in people ≥60 years old, both folate and vitamin B12 shown a nonlinear negative association, and this association of folate also exists in the total population. This may be related to the association between large intake of folate and the lower waist circumference (WC) among the elderly population [63], and WC is one of the criteria for defining obesity in the population. Obesity is a complex metabolic chronic disease, usually accompanied by excessive generation of free radicals and oxidative stress in the vascular endothelium, which leads to an increase in blood pressure [64]. The inconsistency of these research results may be related to the bidirectional effect of metabolic homeostasis: Increased intake of vitamin B6, vitamin B12 and folate can reduce oxidative stress and lower blood pressure by promoting Hcy metabolism [65], B12 acts as a coenzyme for methionine synthase and participates in the process of converting Hcy into methionine through re-methylation. Low serum B12 level was associated with elevated Hcy levels, and elevated Hcy levels have been proven to increase the risk of hypertension through oxidative stress and endothelial dysfunction. Young people (aged 20−39) with high red meat intake (containing dietary B12 and saturated fat) may induce vascular inflammation through the TLR4/NF-κB pathway, strengthening the positive correlation between B12 and hypertension [66]. A recent cohort study shown that people with high intake of animal protein had a positive correlation with blood pressure [67]. High-dose B12 supplementation may interfere with folate metabolism, leading to the accumulation of unmetabolized folate (UMFA), activating the IL-6/TNF-α pathway, and counteracting its blood pressure-lowering effect [68]. Young people consume more red meat than middle-aged and elderly people. Therefore, the positive correlation between vitamin B12 and hypertension is more evident in young people. The linear positive correlation between folate intake and hypertension in the 60 years old population suggests the special metabolic characteristics of the elderly population that the decline in liver metabolic capacity in the elderly population may lead to the accumulation of non-metabolic folate (UMFA), activating pro-inflammatory pathways (such as IL-6/TNF-α), thereby increasing systolic pressure in the population [69]. Moreover, this may also be related to the activity of vitamin B12-dependent methionine synthase. Folate supplementation in a low B12 state may induce “methyl trap” dependence [70]. Furthermore, since the hypertension variable used in the logistics regression comes from “whether the doctor has ever told you about hypertension” and the definition of three consecutive blood pressure measurements exceeding the standard value, it is possible that people whose blood pressure has already returned to normal but were previously diagnosed as having hypertension will be classified into the hypertension group. This group of people, knowing that they have hypertension, consume more nutrients that can control blood pressure, such as B12, thereby showing a positive correlation between vitamin B12 intake and hypertension. In the RCS model, we demonstrated a negative correlation between vitamin B12 intake and diastolic pressure. This may be more reflected in the effect that hypertensive patients have a decrease in diastolic pressure after taking high doses of B12.

## 5. Advantages and disadvantages

We utilized authoritative national research data from the United States and applied a restricted cubic spline (RCS) model to analyze the complex relationship between B vitamins and blood pressure. Revealing the often-obscured nonlinear dose-response relationship in traditional linear models through the use of RCS models. It can clearly uncover subtle associations between research variables, such as threshold effects, U-shaped curves, and saturation points, which cannot be detected using traditional linear regression. Specifically, it enables us to determine the precise intake level at which the protective effect of certain vitamins (such as vitamin B2) on diastolic pressure begins to weaken, and crucially, it highlights the potential risks associated with excessive intake of other vitamins (such as choline and vitamin B12). By doing so, our analysis is not just about confirming associations, but providing actionable insights into the optimal intake window for different age groups, thereby providing precise data support for hypertension prevention and control strategies in specific age groups.

Due to the cross-sectional nature of the study, we were unable to investigate the causal relationship between B vitamins and hypertension, to analyze the causal relationship between intake and blood pressure, and to conduct clinical trials to explore the optimal intake of various vitamins at different ages. Because the data is from the United States, the dietary patterns of this group (such as fortified foods) may vary globally, and the intake of fortified foods may overestimate the impact of B vitamins on blood pressure. However, the probability of consuming fortified foods among the general population is relatively low, so the results cannot be generalized to other groups and races.

Our research also has limitation in the classification of supplements and their synergistic effects. The NHANES data cannot distinguish between multivitamins and single B-vitamin supplements, and does not take into account the confounding effects of supplements, which may overlook the synergistic or antagonistic effects between nutrients. For instance, excessive intake of a single vitamin supplement may mask toxic signals, while a multivitamin may exaggerate the synergistic effects. For example, the antioxidants in a multivitamin (such as at-soluble vitamins (A, E, D, and K) [71] may indirectly interfere with the association between B vitamins and blood pressure by regulating oxidative stress, in the elderly population, the negative correlation between B vitamins and blood pressure may be overstated due to the presence of calcium/magnesium in various vitamins and other independent blood pressure-lowering components. Moreover, our independent analysis of B vitamins may overlook synergistic interactions critical to their biological functions.

Secondly, there may also be measurement errors and residual confounding. The dietary vitamin intake was obtained through questionnaire surveys and telephone follow-ups, which introduced measurement errors. The daily variations in the 24-hour recall may weaken the true dose-response relationship at 0, especially in terms of threshold effects; obese participants may incorrectly report consuming high-calorie/ vitamin B-rich foods, thereby distorting the association between high intake and hypertension; changes in the survey date and differences in intake between weekdays and weekends further reduced accuracy. Residual confounding factors caused by unmeasured variables still exist, such as genetic polymorphisms may amplify the association between vitamin B12 and hypertension, while environmental exposure is related to endothelial dysfunction and may alter the effect of vitamins.

The B6/B9/B12 triad regulates the clearance of Hcy through the methylation (dependent on B12) and transsulfurization (dependent on B6) pathways. However, our hierarchical model may underestimate these effects due to unmeasured confounding factors (methylmalonic acid). Vitamin B2 promotes the regeneration of glutathione, while B6 inhibits the generation of ROS by activating superoxide dismutase. Their combined antioxidant capacity reduces oxidative stress markers by approximately 30% compared to their individual effects, which may explain the stronger L-type association in elderly multivitamin users. However, advanced methods such as weighted quantile sum (WQS) regression or principal component analysis (PCA) cannot be used due to the limitations of the NHANES data: 1. Unidentifiable interactions-- Reverse causal relationships (dietary changes driven by hypertension) confound the aggregation patterns of nutritional components, violating the assumptions of PCA. 2. Supplementation heterogeneity-- The components of multiple vitamin preparations vary greatly (containing minerals with independent blood pressure-lowering effects such as magnesium), which leads to unquantifiable biases in the assessment of interactions. Future research will need to further combine metabolomics (SAM/SAH ratios) and factor experiments to validate these interactions.

Moreover, homocysteine and anemia depend on the regulation of B vitamins (for example, B6/B9/B12 in homocysteine metabolism) and are related to hypertension. However, our data cannot explore the association between vitamins, homocysteine, and anemia, and thus cannot understand whether homocysteine and anemia will affect the association between B vitamins and blood pressure. Future research should include biomarkers (such as serum folate levels) to verify the intake and address these limitations.

## 6. Conclusion

This cross-sectional study involving 3654 American adults found a significant association between the intake of B vitamins in the diet and blood pressure results across different age groups. In all age groups, the diastolic pressure shown a linear decreasing trend. There are non-linear dose-response relationship between B vitamins intake and blood pressure. This suggests that the current one size fits all dietary recommendations for B vitamins may not be the best choice for blood pressure outcomes throughout adulthood. Our data suggests that nutritional needs and responses may exhibit non-linear changes with age. The sustained negative correlation between different age groups and diastolic blood pressure reinforces the existing policy of promoting adequate intake of B vitamins as part of a heart healthy diet.

However, since this is a cross-sectional analysis, it is impossible to infer causality, and even after adjusting for demographic characteristics, lifestyle, and comorbidities, residual confounding factors may still persist. Longitudinal studies are needed to verify these associations, especially for those vitamins that shown a reversed trend with age.

Future studies could explore whether personalized intake thresholds for B vitamins can be determined based on the nonlinear dose-response pattern, in order to optimize blood pressure outcomes for different age groups. Meanwhile, mechanistic studies should investigate the biological basis of these age-specific effects. Before obtaining this evidence, clinical and public health work should continue to emphasize achieving the recommended intake of B vitamins through a balanced diet rich in fruits, vegetables, and whole grains, as blood pressure is regulated by age.

## Supporting information

S1 Fig The no relationship between B vitamins intake and hypertension by weighted RCS model. (TIF)

S2 Fig The no relationship between B vitamins intake and systolic pressure by weighted RCS model. (TIF)

S3 Fig The no relationship between B vitamins intake and diastolic pressure by weighted RCS model. (TIF)
