# Supplementary figures and images for: Association between dietary B vitamins intake and age-specific blood pressure: A cross-sectional study in American adults
Source: PLoS One. 2025 Oct 22;20(10):e0335306. doi: 10.1371/journal.pone.0335306 (PMC12543131; doi:10.1371/journal.pone.0335306)

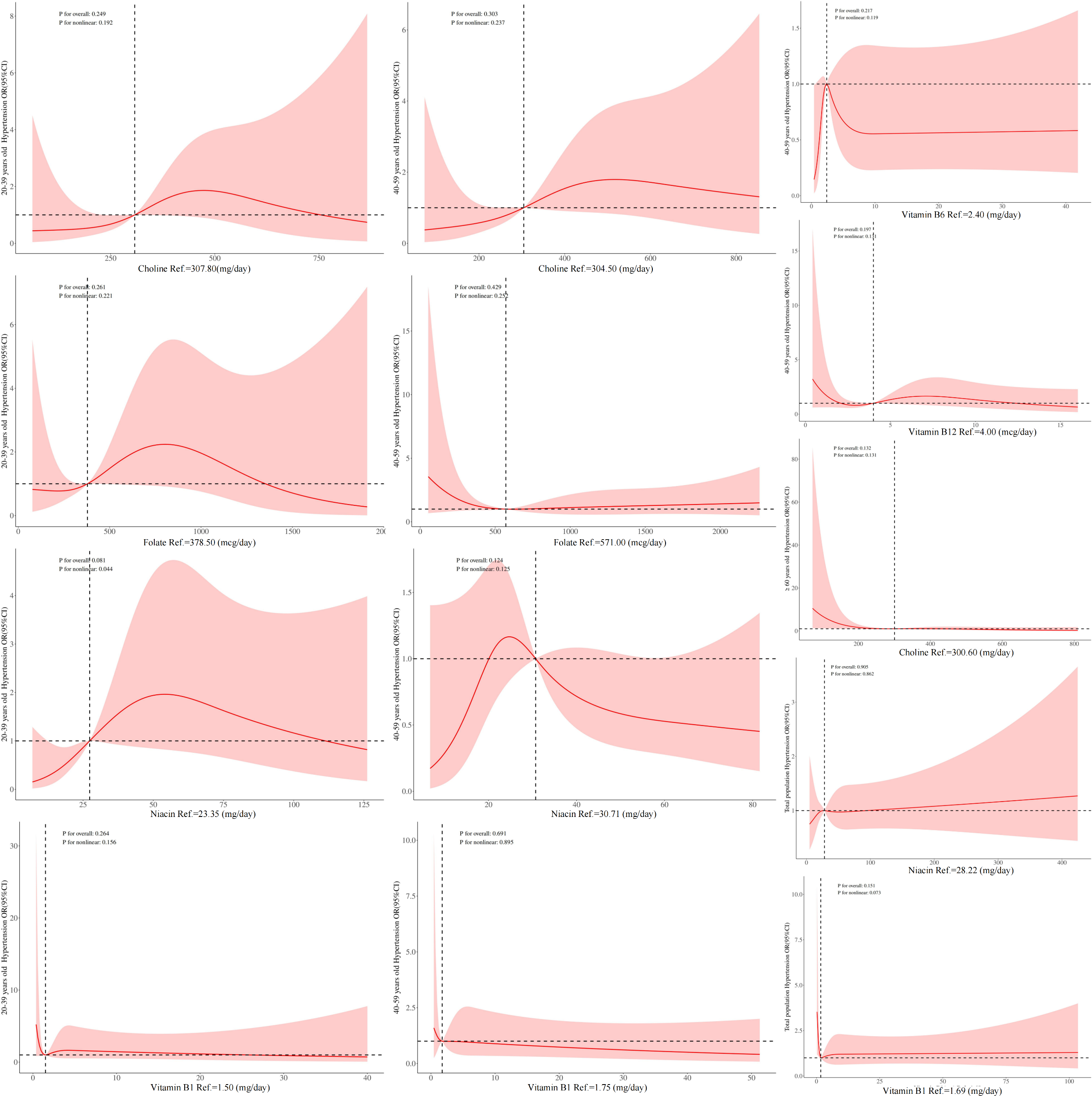

Supplement: S1 Fig — (TIF) [file pone.0335306.s001.tif]

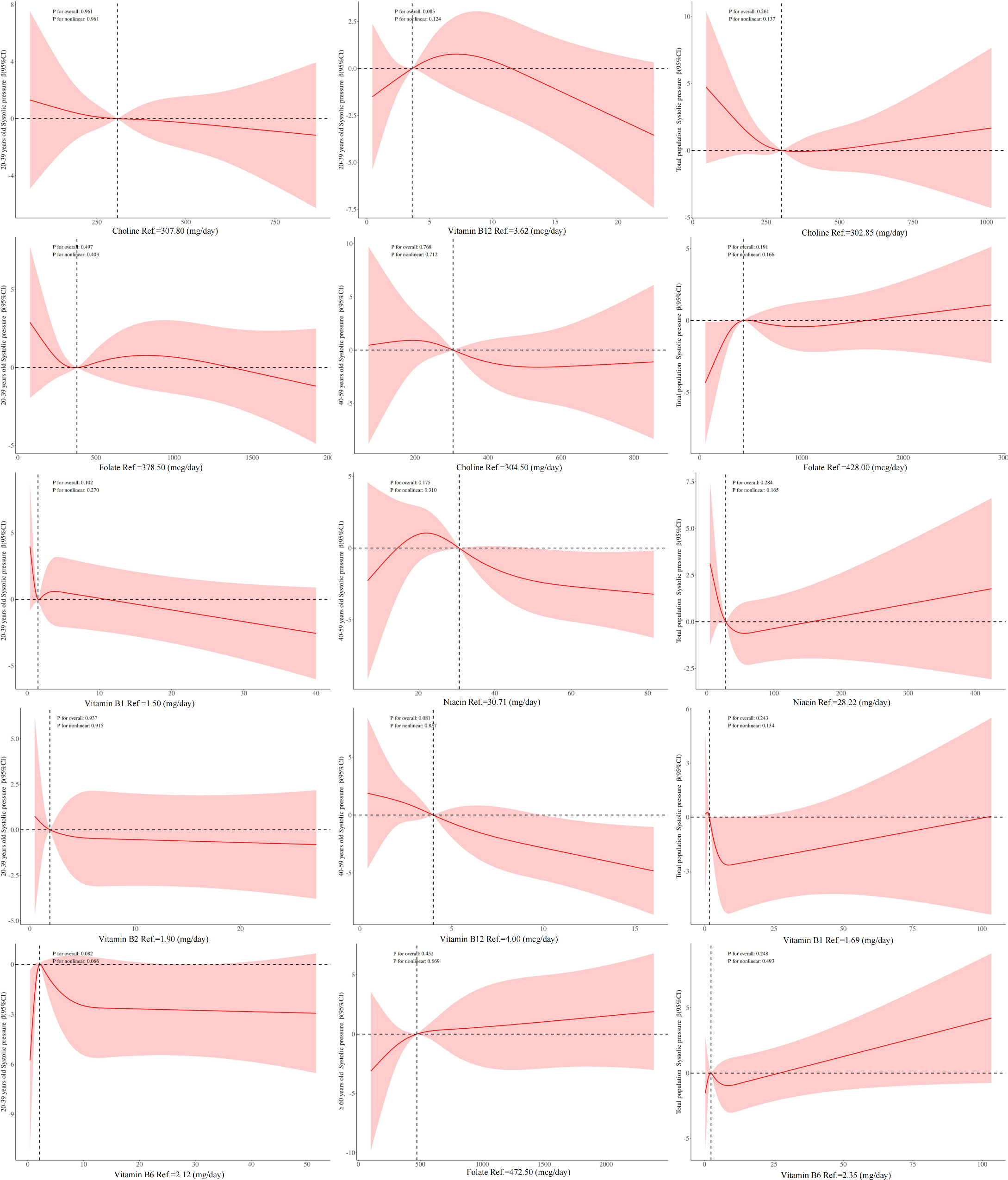

Supplement: S2 Fig — (TIF) [file pone.0335306.s002.tif]

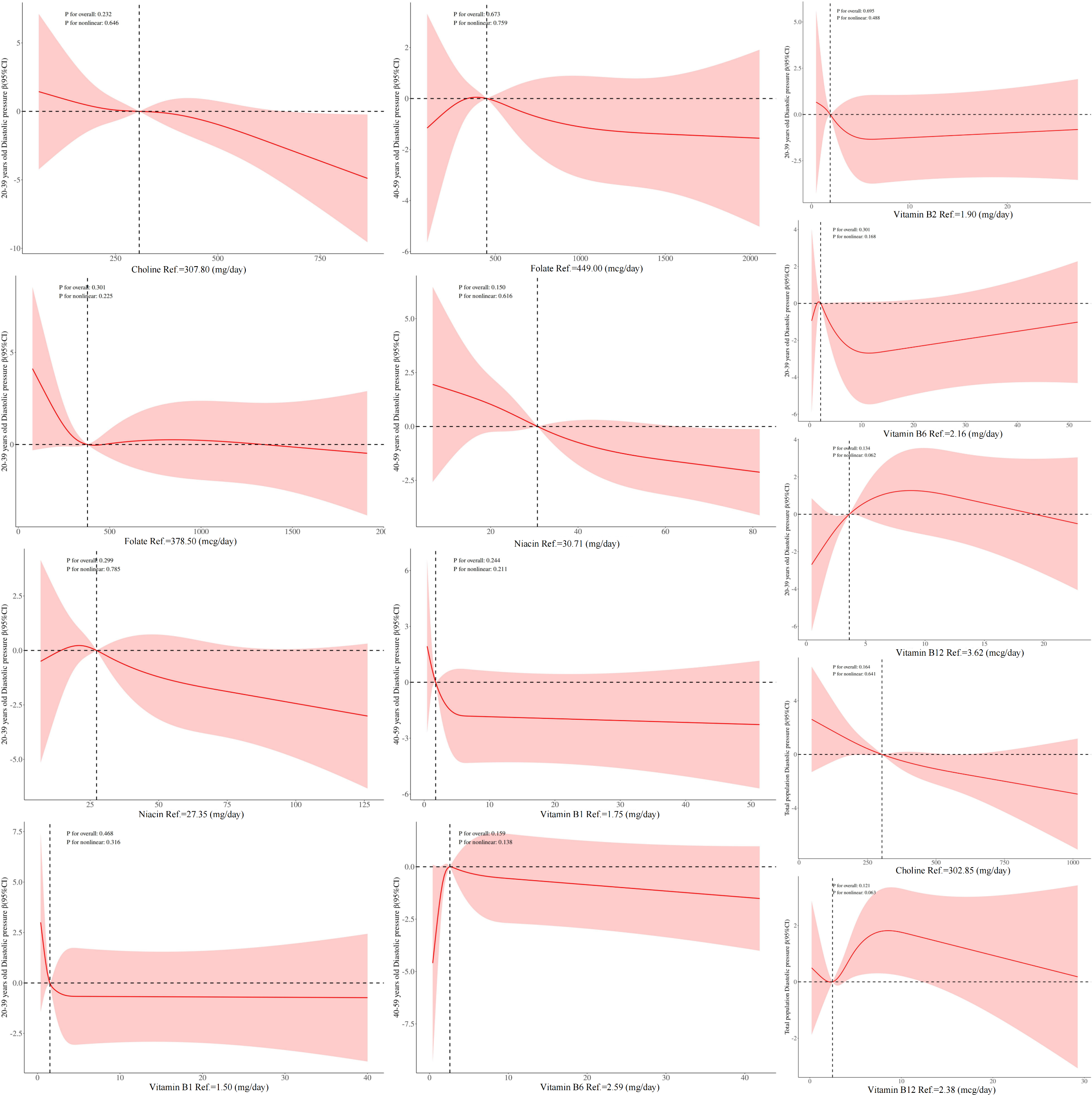

Supplement: S3 Fig — (TIF) [file pone.0335306.s003.tif]
